# Treating a GAD65 Antibody-Associated Limbic Encephalitis with Basiliximab: A Case Study

**DOI:** 10.3389/fneur.2015.00167

**Published:** 2015-08-03

**Authors:** Guido Widman, Kristin Golombeck, Hubertus Hautzel, Catharina C. Gross, Carlos M. Quesada, Juri-Alexander Witt, Elena Rota-Kops, Johannes Ermert, Susanne Greschus, Rainer Surges, Christoph Helmstaedter, Heinz Wiendl, Nico Melzer, Christian E. Elger

**Affiliations:** ^1^Department of Epileptology, University of Bonn, Bonn, Germany; ^2^Department of Neurology, University of Münster, Münster, Germany; ^3^Department of Nuclear Medicine (KME), Medical Faculty, Research Center Jülich, Heinrich-Heine-University Düsseldorf, Jülich, Germany; ^4^Department of “Neurowissenschaften und Medizin” 4 (INM-4), Research Center Jülich, Jülich, Germany; ^5^Department of Radiology, University of Bonn, Bonn, Germany

**Keywords:** limbic encephalitis, GAD65, epilepsy, basiliximab, cytotoxic T lymphocytes

## Abstract

**Background:**

Antibodies (ABs) against the 65-kDa isoform of the intracellular enzyme glutamate decarboxylase (GAD65) have been found in limbic encephalitis (LE) and other neurological conditions. The direct significance of anti-GAD65-ABs for epilepsy is unclear. However, in histological preparations from biopsies of resective epilepsy surgeries, predominantly cytotoxic T-lymphocytes were detected making close contacts to neurons. Activated T-lymphocytes can, in turn, be selectively controlled by therapeutic interleukin-2 receptor Abs, such as basiliximab.

**Case presentation:**

We report of a 25-year-old male patient with epilepsy since the age of 18 and displaying clinical signs of LE and a high titer of GAD65 ABs in cerebrospinal fluid (CSF) and serum. Monthly, repetitive, intravenous cortisone pulse therapies that were initially administered for 6 months failed to improve his condition. Subsequent flow-cytometry analysis of CSF showed especially an increased fraction of activated HLA-DR^+^ CD8^+^ T-lymphocytes (fCD8^+^TL) when compared to controls. Thus, a second, intravenous cortisone pulse therapy with an additional basiliximab dose of 20 mg/month was started. After 3 months, the fCD8^+^TL in the CSF normalized; after 6 months, the psychological impulse-control deficits normalized; and after 11 months the patient was seizure free. However, 7 weeks later, seizures and, later on, psychological deficits recurred and fCD8^+^TL was once again present in the CSF. Flumazenil PET, magnetic resonance imaging-volumetry, and neuropsychological changes during therapy are described.

**Conclusion:**

The correlation of the fCD8^+^TL in the CSF with clinical and paraclinical measures of disease activity combined with the unambiguous response to basiliximab strongly argues in favor of the putative pathogenic role fCD8^+^TL in anti-GAD65 LE. The clinical relapse at the end of the observation period might be due to the formation of human anti-drug ABs, a well-known complication of therapy with chimeric ABs.

## Introduction

**Figure 1:**
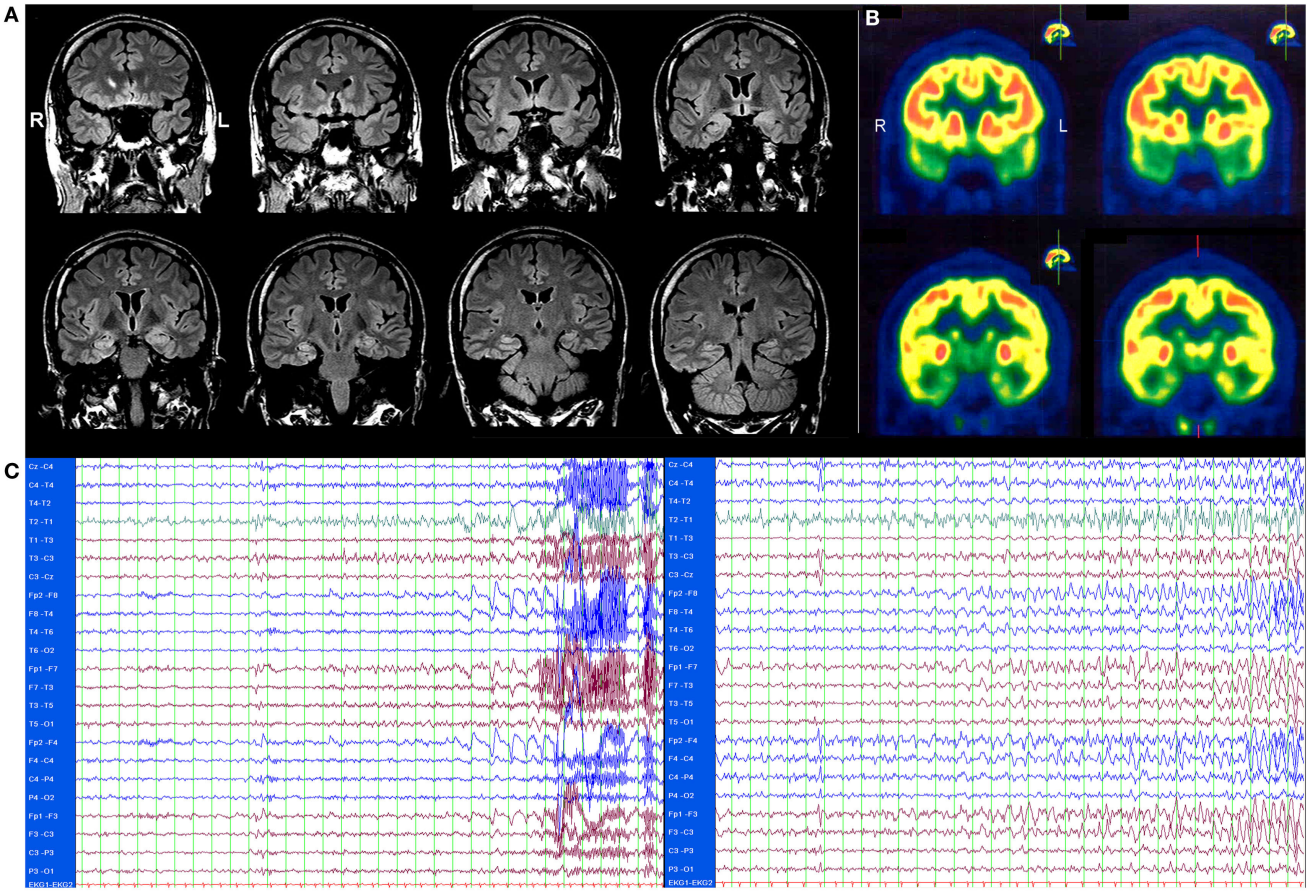
**(A)** Brain magnetic resonance imaging (MRI), T2/fluid attenuated inversion recovery (FLAIR) showing a distinct bilateral temporomesial hyperintensity without atrophy. **(B)** 2-fluoro-2-desoxy-d-glucose positron emission tomography (FDG-PET) showing a bitemporal (right > left), hypometabolism. Both images were scanned before therapy, in November 2011. **(C)** EEG of two seizures. One second per line. Left: seizure starting with atypical sharp-slow-wave complex and irregular theta activity left temporal. Clinical start 11 s later with eye opening, oral automatisms, leg movements, later on secondary generalization. Right: seizure starting with irregular delta activity right frontotemporal with fast transition to left frontal contacts. Clinical start 34 s later with eye opening, non-verbal vocalization, sitting up, staring, later on secondary generalization.

In 2009, a male patient with temporal lobe epilepsy (TLE) that started at the age of 18 was admitted to the Department of Epileptology, University of Bonn, 2 years after his initial diagnosis. He displayed some clinical signs compatible with limbic encephalitis (LE): seizures of temporal semiology starting in adult age but lasting no longer than 5 years, as well as affective disturbances with prominent mood lability, or disinhibition (1, 2). Since symptomatic, anticonvulsant therapy failed to control his seizures, the patient was assessed in 2011 to ascertain whether or not he was a suitable candidate for epilepsy surgery. Brain magnetic resonance imaging (MRI) revealed distinct signs of temporomesial encephalitis [T2/fluid attenuated inversion recovery (FLAIR): distinct hyperintensity without atrophy, see Figure 1A]. A 2-fluoro-2-desoxy-d-glucose positron emission tomography (FDG-PET) displayed a bitemporal (right > left), hypometabolism (see Figure 1B). Neuropsychological tests [EpiTrack^®^ (3), VLMT (4), and DCS-R (5)] revealed deficits in frontal and right temporal functions. During scalp video-EEG monitoring, three habitual seizures were recorded: two seizures originated from the left temporal region and one seizure from the right frontotemporal region (see Figure 1C). Because of the bilateral seizure onset and the lack of a clear epileptogenic lesion in the MRI (e.g., unilateral hippocampal sclerosis), the presurgical evaluation was discontinued. Furthermore, complementary investigations including the search for autoantibodies against neuronal antigens in the serum and later on cerebrospinal fluid (CSF) were performed (Euroimmun AG, Lübeck, Germany): the serum was tested for BIOCHIP-Mosaic immunofluorescence Anti-Hu, -Ri, -Yo, -Tr, -MAG, -Myelin, -Ma/Ta, -Amphiphysin, -CV-2, -Aquaphorin-4, -Glycinereceptors, -GAD (65 kDa); IgAGM IFT Anti-NMDA, -AMPA, -GABA-b receptors; IgAGM IFT Anti-LGI1, -CASPR2; Anti GAD (65 kDa) IgG ELISA, Anti potassium-channels RIA.

A high concentration of glutamic acid decarboxylase (65 kDa)-antibodies (GAD65-ABs) was found in serum (>2000 IU, ELISA; 1:1000 BIOCHIP-Mosaic immunofluorescence) and later on in the CSF (Biochip immunofluorescence++ 1:10), leading to the final diagnosis of a GAD65-AB positive LE. All other tests for competing causes of mediotemporal encephalitis were normal. Importantly, other conditions associated with GAD65 ABs (such as type 1 diabetes mellitus) were absent in our patient.

During the patient’s in-hospital stay, a postictal episode with acute physical aggression occurred. Later on, repeated episodes of seizure independent, inappropriate behavior with physical and verbal aggression were observed. Finally, the patient started arguing with another fellow patient about his supposed loud talking, which later resulted in a violent assault. Consequently, the patient was then transferred to a locked psychiatric ward and an antipsychotic therapy with risperidone was started.

Additionally, a cortisone pulse therapy with a monthly application of 1 g/day methylprednisolone (Urbason^®^, Sanofi) over 5 days was administered for 6 months. This therapy, however, did not change his disease in any way. The time course of the disease can be seen in Table 1.

**Table 1:** **Time course of disease**.

Visit (date of discharge)	Basiliximab 20 mg	Cortisone pulse	Patient status	Seizures	Anti-epileptic drugs	Other medications	EEG	Other events	Gad65 serum IgG Elisa (IU/ml) IgG IFT titer	Gad65 CSF IgG IFT Titer	OKB CSF	FACS
12.01.07			I/e	GTC: 1/m	Initially PHT 200/d + LTG, later: LEV 500-0-1000 LTG 200-0-250	Escitalopram 10-0-0						
						Opipramol 50-0-0						

20.10.09			O	Seizure free 3 m	LEV 1000-0-1000 LTG 100-0-100	Escitalopram 20-0-0	Theta left temporal					

18.09.10			O/e	CPS 1/m	LEV 2000-0-2000 LTG 100-0-100	Escitalopram 20-0-0	Normal					

31.03.11			O/e	CPS 1/m	LTG 100-0-100		Theta bi-temporal l > r					

11.04.11			I/e	GTC-series	LTG 150-0-150		Theta bi-temporal l > r					

23.05.11			I/e	GTC-series	LTG 200-0-200, VPA 300-0-300 LEV 500-0-500		Diffuse slowing					

25.08.11			I/e	3-4 CPS/m	LTG 200-0-200, VPA 300-0-300 LEV 500-0-500							

09.09.11			O	10 CPS/m	LEV 1000-0-1000 LTG 200-0-200							

24.11.11			I	2 CPS/m	LTG 200-0-200, OXC 600-0-600 PGB 0-0-75	Olanzapine 0-0-2.5	TIRDA l > r, SSW in sleep	FDG-PET	>2000 n.d.			
				1 GTC/m								

14.12.11		5 d/month, 6 months	I/e	2 CPS/m	LTG 200-0-200, OXC 600-0-600 PGB 0-0-75	Risperidone 1-0-0						
				1 GTC/m								

19.12.11			O	2 CPS/m	LTG 200-0-200, OXC 600-0-900 PGB 0-0-75	Risperidone 1-0-0						
				1 GTC/m								

08.02.12			I	3 CPS/m	LTG 200-0-200, OXC 600-0-600 PGB 0-0-75	Risperidone 1-0-0			>2000 1:1000	1:10	IgG	
				1 GTC/m								

14.07.12		I	2 CPS/m	LTG 200-0-200, OXC 600-0-600 PGB 0-0-75	Risperidone 1-0-0	SSW r. temporal		>2000 1:1000			
			1 GTC/m								

13.03.13			O	2 CPS/m	LTG 200-0-200 OXC 750-0-750 PGB 150-0-150	Risperidone 1-0-0						
				1 GTC/m								

11.04.13			I	2 CPS/m	LTG 150-0-150 OXC 600-600-600	Risperidone 1-0-0	SSW r. temporal		>2000 1:1000			
				1 GTC/m								

11.05.13			O/e	2–3 CPS/m	LTG 100-100-100 OXC 600-600-600 ZON 200-0-200	Risperidone 1-0-0						
				1 GTC/m								

01.08.13	#1	5 d	I	2–3 CPS/m	LTG 100-100-100 OXC 600-600-600 ZON 200-0-200	Risperidone 0.5-0-0		Flumazenil PET #1	>2000 1:320	1:3.2	–	Before therapy
				1 GTC/m								

03.09.13			I/e		LTG 100-100-100 OXC 600-600-600 ZON paused	Risperidone 0.5-0-0		renal failure				

17.09.13	#2	5 d	I	CPS 3/m	LTG 100-100-150, OXC 600-600-600 ZON 200-0-200	Risperidone 0.5-0-0	SSW r. temporal					

31.10.13	#3	5 d	I	CPS 5/m	LTG 100-100-150, OXC 600-600-600 ZON 200-0-200	Risperidone 0.5-0-0			n.d. 1:100	Neg.	–	After therapy

12.11.13	–		O/e		LTG 100-100-150, OXC 600-600-600 ZON 200-0-200	Risperidone 0.5-0-0	bitemporal slowing l > r					

09.12.13	#4	5 d	I	CPS 3/m	LTG 100-100-150, OXC 600-600-900 ZON 200-0-200	Risperidone 0.5-0-0	SSW r. temporal		n.d. 1:1000			

24.01.14	#5	5 d	I	7 CPS/m	LTG 100-100-150, OXC 600-600-900 ZON 200-0-200	Risperidone 0.5-0-0		FDG-PET				
				2 GTC/m								

12.03.14	#6	5 d	I	CPS 3/m (only nightly, shorter)	LTG 100-100-150, OXC 600-600-900 ZON:weight-loss	Risperidone 0.5-0-0	SSW bi-temporal r >> l	Flumazenil PET #2	n.d. 1:320	Neg.	–	Before therapy

19.04.14	#7	3 d	I	CPS 2/m (only nightly, shorter)	LTG 100-100-150, OXC 600-600-900 weight-gain after removal of ZON	Risperidone stopped						

30.05.14	#8	3 d	I	7 CPS/m	LTG 100-100-150, OXC 600-600-900 PER 0-0-2							
				1 GTC/m								

14.07.14	#9	3 d	I	CPS 3/m	LTG 100-100-150, OXC 600-600-900 PER 0-0-8							

15.08.14	#10	3 d	I	CPS 3/m	LTG 100-100-150, OXC 600-600-900 PER 0-0-10							

10.09.14	#11	3 d	I	CPS 1/m	LTG 100-100-150, OXC 600-600-900 PER 0-0-10							

27.10.14	#12	3 d	I	No seizures	LTG 100-100-150, OXC 600-600-900 PER 0-0-10				n.d. 1:1000			

06.12.14	#13	3 d	I	CPS 3/m (7 weeks seizure free)	LTG 100-100-150, OXC 600-600-900 PER 0-0-10							

19.01.15	#14	3 d	I	CPS 3/m	LTG 100-100-150, OXC 600-600-900 PER 0-0-10				n.d. 1:1000	1:1	–	After therapy

*I, inpatient; O, outpatient; /e, external hospital; d, days; CPS, complex partial seizures; GTC, generalized tonic clonic seizures; m, month; PHT, phenytoin; LTG, lamotrigine; VPA, valproate; LEV, levetiracetam; OXC, oxcarbazepine; ZON, zonisamide; PER, perampanel; r, right; l, left; TIRDA, temporal intermittent rhythmic delta activity; SSW, sharp-slow-waves; n.d., not done*.

The subject of this case report is the second immunomodulatory therapy comprising a cortisone pulse therapy with monthly applications of 1 g/day methylprednisolone (Urbason^®^, Sanofi) over 5 days and 20 mg basiliximab monthly applied on day 3 of the cortisone pulse therapy. The therapy was continued for 6 months under nearly constant anticonvulsant medication and, because of initial improvements, his therapy was extended to 14 months. However, during the extension period, the anticonvulsant medication was changed due to side effects and the cortisone pulse therapy was shortened to 3 days, with an application of 20 mg basiliximab on day 2.

## Background

Limbic encephalitis is an autoimmune encephalitis with mediotemporal lobe symptoms caused by inflammatory lesions in limbic structures, which leads to a TLE, impairment of recent memory, and affective disturbances, which have mostly been observed in adults. Initially, LE was thought to mainly occur as a paraneoplastic symptom. However, it can also occur as a non-paraneoplastic disease (2). LE is subclassified according to the presence of autoantibodies against either neuronal surface antigens or intracellular targets (6). In a series of nine patients with LE symptoms and high titer GAD65 ABs, none of the seven patients who received intravenous Methylprednisolone pulses (500–1000 mg/day on three to five consecutive days) for up to 6 months became seizure free about 12 months after their initial assessment (1). Therapy was even escalated in some cases and a high dosage of intravenous immunoglobulin or cyclophosphamide was administered. However, these interventions resulted in the concentration of ABs being reduced to only 57% (69–3%) of the initial concentration.

The histopathology, which was available, revealed that T-lymphocytes, in particular, were found in patients with GAD65-AB LE (1, 7).

A possible therapy aimed at reducing the amount of activated T-lymphocytes can be initiated by using therapeutic ABs against the activated interleukin-2 receptor (8). Up until that point in time, only basiliximab (Simulect^®^, Novartis International AG) was available. Basiliximab is a chimeric, mouse-human monoclonal AB to the α chain (CD25) of the interleukin-2 receptor of T-lymphocytes. It was approved in 1998 to prevent rejection in organ transplantation, especially in kidney transplants. In this case, it was given as an individual treatment attempt after written informed consent was obtained. The therapy included monthly intravenous applications of 20 mg basiliximab. Since basiliximab is a chimeric AB, which carries a significant risk for the development of anaphylactic reactions due to human anti-drug antibodies (HADAs), (9–11) and also because it is recommended that basiliximab be combined with additional immunotherapies (12), an additional cortisone pulse therapy with monthly applications of 1 g Methylprednisolone (Urbason^®^) per day over 5 days was applied. Basiliximab was given on day 3 of the cortisone pulse therapy in order to achieve maximum protection against anaphylactic reactions due to HADAs. As described above, the therapy was continued for 6 months and, because of initial improvements, it was then extended to 14 months. During the extension period, the anticonvulsant medication had to be changed due to side effects and the cortisone pulse therapy was shortened to 3 days, with an application of 20 mg basiliximab on day 2.

## Results

In the presented case, GAD65-ABs were above the limit of 2000 IU before, during, and after initial Cortisone pulse therapy in the peripheral blood (PB). In the CSF, GAD65-ABs were also found during and after cortisone pulse therapy. The first examination of CSF did not occur prior to the start of cortisone pulse therapy, since hospitalization was abruptly terminated by transferring the patient to a locked psychiatric ward.

**Figure 2:**
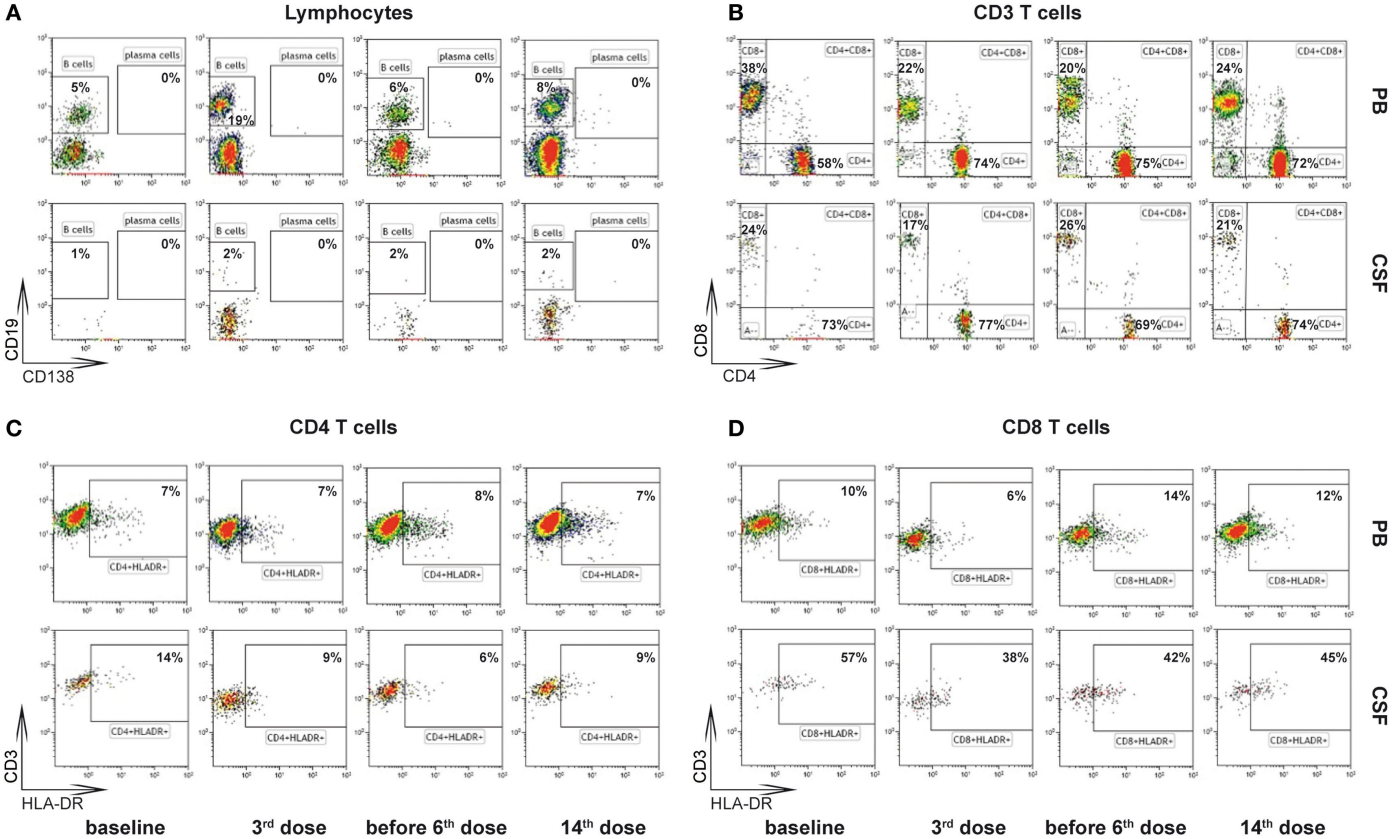
**Gating strategy of flow-cytometry (13)**. Cells from peripheral blood (PB) and cerebrospinal fluid (CSF) at baseline, after 3rd dose, before 6th dose, and after 14th dose of basiliximab were stained using fluorochrome labeled antibodies directed against the linage markers CD45 (leukocytes), CD14 (monocytes), CD19 (B cells), CD138 (CD19^low^CD138^+^ plasma cells), CD3 (T cells), CD56 (NK cells), CD4 (CD4^+^ T cells), and CD8 (CD8^+^ T cells) and the activation marker HLA-DR and analyzed by multicolor flow cytometry. CD45^+^ leukocytes were selected in a CD45-KromeOrange versus forward scatter (FSC) plot. CD45^+^ cells were then displayed in a CD14-FITC versus sideward scatter (SSC) plot and CD14^−^ lymphocytes were selected. **(A)** Lymphocytes were displayed (CD138-PE/CD19-A700) and CD19^+^CD138^−^ B cells or CD19^low^CD138^+^ plasma cells were selected. **(B)** Lymphocytes were displayed (CD3-PC5.5/CD56-PC7 plot) and CD3^+^CD56^−^ T-cells were selected. T-cells were split into CD4^+^ and CD8^+^ subsets in a CD4-APC CD8-PacificBlue plot. **(C,D)** The percentage of HLA-DR positive CD4^+^ T-cells **(C)** and CD8^+^ T-cells **(D)** was analyzed including CD45^+^CD3^+^CD56^−^CD4^+^CD8^−^ and CD45^+^CD3^+^CD56^−^CD4^−^CD8^+^ lymphocytes, respectively.

**Figure 3:**
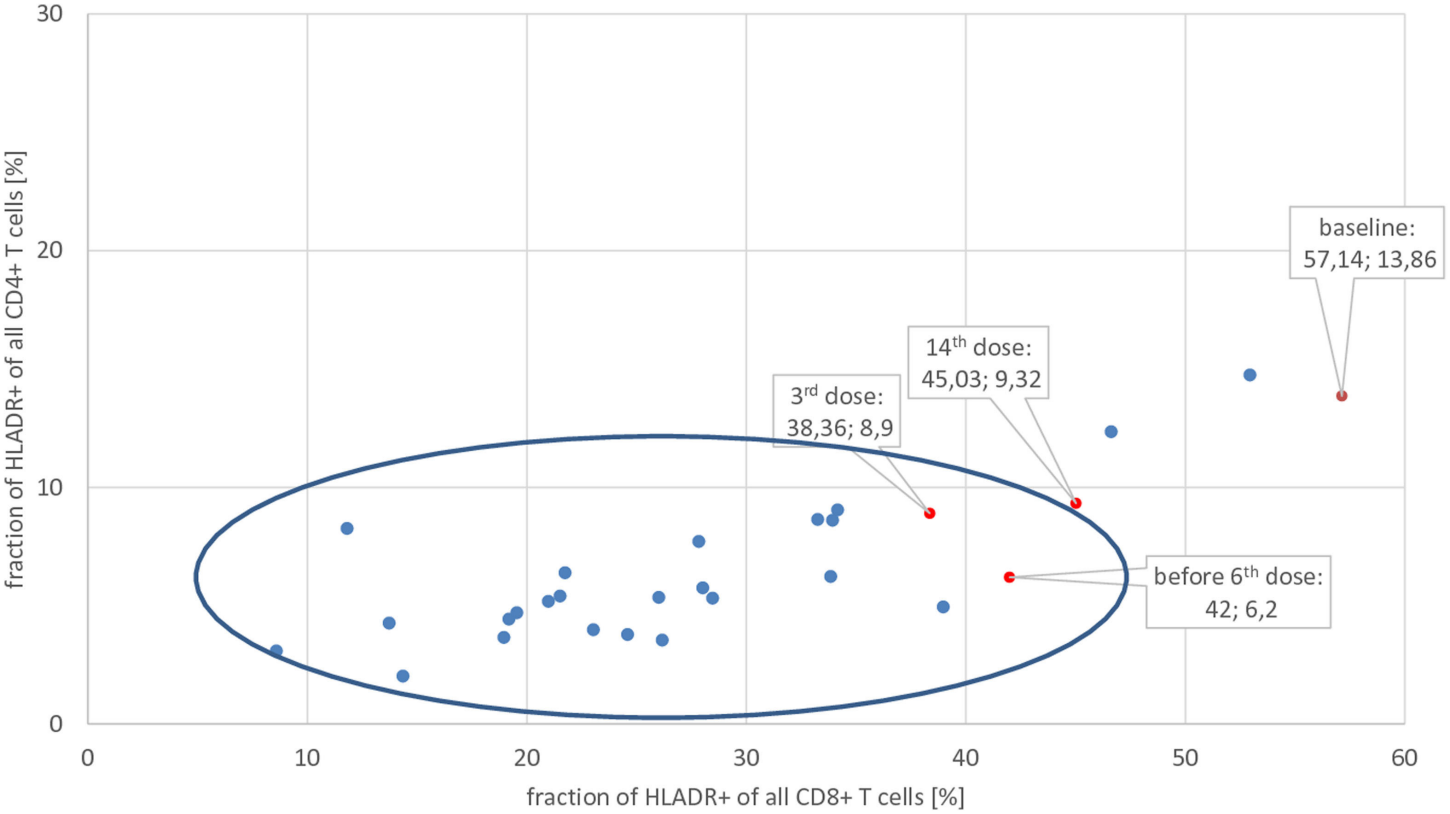
**Fraction of activated (**=**HLA-DR^+^) CD8^+^ and CD4^+^ T-lymphocytes of the patient at baseline, after 3rd, before 6th, and after 14th application of basiliximab (red dots), compared with normal controls (blue dots)**. Only data from CSF are shown. The center of the blue ellipse is the mean of normal controls; the major axis of the ellipse represents mean plus/minus 2 SDs of the fraction of activated CD8^+^ T-lymphocytes; the minor axis of the ellipse represents mean plus/minus 2 SDs of the fraction of activated CD4^+^ T-lymphocytes of normal controls. Thus, dots outside of this ellipse significantly differ from normal controls.

In order to evaluate whether or not there are predominantly activated T-lymphocytes in the central nervous system in this patient, like histopathological findings showed in former patients with GAD65-AB LE, flow-cytometry analysis of PB and CSF was performed using a 10 color panel (Figure 2, “baseline,” see also Changes in the CSF Using Flow Cytometry and Antibody Tests) in order to differentiate granulocytes, monocytes, NK-cells, NKT cells, B-lymphocytes (plasma-cells), and T-lymphocytes (CD4/CD8 and the stimulation-marker HLA-DR) (13). Results were compared with 24 controls (emergency-patients, mostly with severe headache, but ultimately without evidence for meningitis or subarachnoid bleeding, see Figure 3 “baseline”). As compared to normal controls, the fraction of activated CD4^+^ as well as CD8^+^ T-lymphocytes was significantly increased (by more than 2 SDs) in the CSF.

In order to monitor the course of disease during therapy, repeated neuropsychological tests, MRI volumetry, measures of AB-titers, and seizure counts were performed. GAD65 is an enzyme that catalyzes the decarboxylation of glutamic acid to gamma-aminobutyric acid (GABA). It can be expected that if this disease implies a dysfunction of GABAergic neurons, it might also reduce the amount of available GABA in the brain and therefore increase the number of GABA-receptors. This should result in more (GABA_A_) binding sites, e.g., for 11C-flumazenil PET. Therefore, before and after 6 months of therapy, an 11C-flumazenil PET was performed in order to achieve an additional biomarker to evaluate if a specific effect existed, which justified a continuation of the therapy.

### Changes in the CSF using flow cytometry and antibody tests

Cells derived from the PB and the CSF of the patient with GAD65-AB-associated autoimmune encephalitis at baseline, after the 3rd dose, before the 6th dose, and after the 14th dose of basiliximab were analyzed using the flow cytometry, as described in Figure 2.

The percentage of activated cytotoxic T-lymphocytes (HLA-DR^+^ CD8^+^) and T-helper cells (HLA-DR^+^ CD4^+^) was compared with 24 controls (emergency patients, mostly with severe headache, but ultimately without evidence for meningitis or subarachnoid bleeding, see Figure 3): before therapy, the fraction of activated CD4^+^ as well as CD8^+^ T-lymphocytes was significantly increased (more than 2 SDs) in this patient’s CSF. After the third application of basiliximab, both parameters were within normal limits and remained there prior to the sixth application. GAD65-ABs were no longer detectable in the CSF after the third and before the sixth application of basiliximab, in contrast to the time before treatment (Table 1). A last measurement was made after the 14th application, about 2 months after seizures recurred. In the two-dimensional plot, the measurement of percentage of activated CD4^+^ and CD8^+^ T-lymphocytes marginally crosses the border of 2 SDs and is once again outside the normal range. In undiluted CSF, the immunofluorescence test for GAD65-AB was positive again.

In CSF, predominantly T-lymphocytes, but only a few B-cells and no plasma cells, can be found. The fraction of B-cells (% of all lymphocytes) was between 0.7 and 1.81% and thus always within normal limits (mean: 0.92%; SD: ±0.83%).

The absolute number of leukocytes in the CSF was always within normal limits. Oligoclonal bands, IgG, just in CSF, were only positive at the first CSF analysis during the first cortisone pulse therapy.

### Changes in neuropsychology, behavior, and EEG

**Figure 4:**
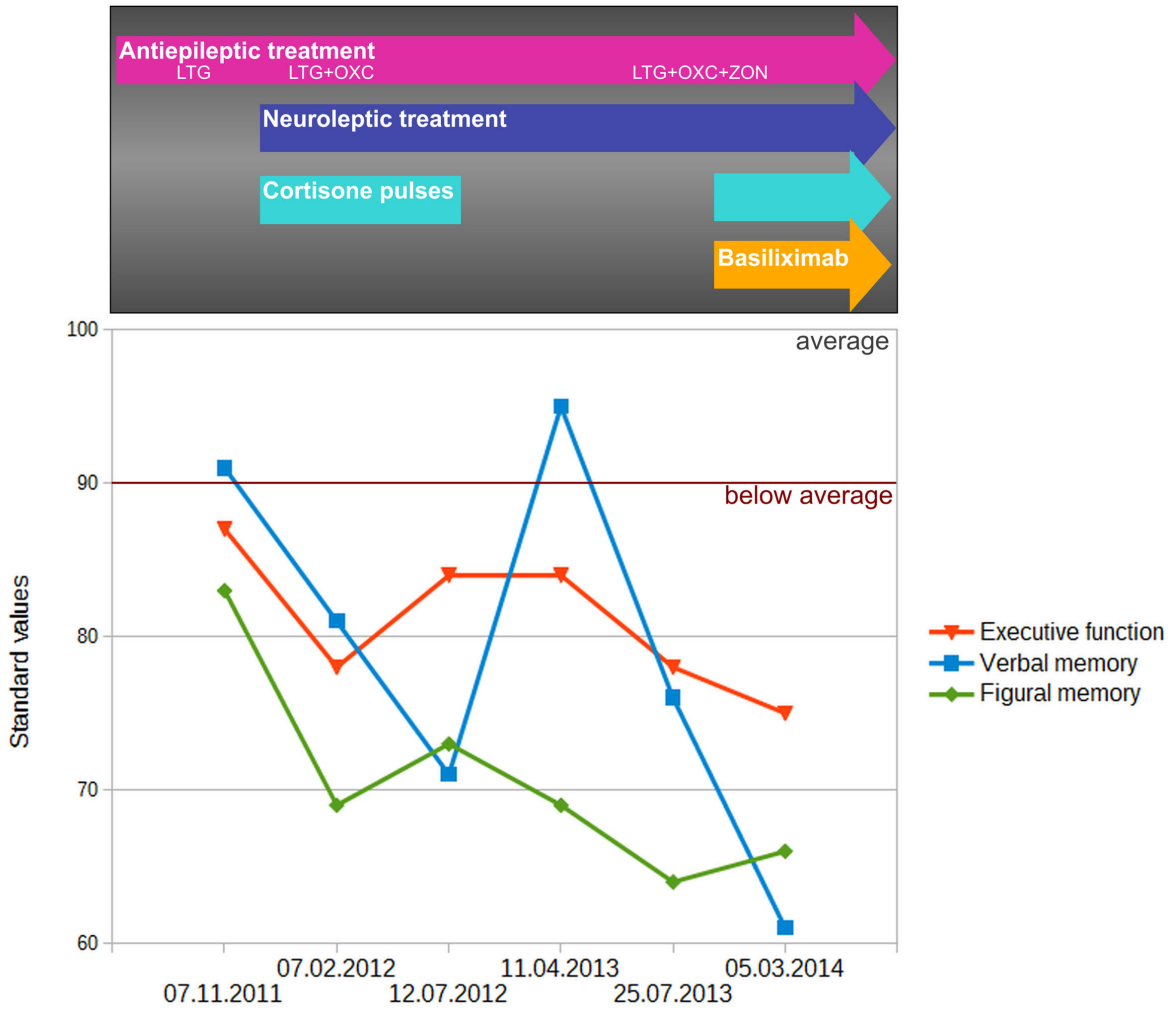
**Change of performance in executive function (EpiTrack^®^) as well as verbal (VLMT delayed free recall after 30 min) and figural (DCS-R learning performance across five trials) memory performance**. Memory performance was standardized (standard values; mean = 100, SD = 10) according to a normative sample of 488 healthy volunteers who underwent both tests for co-normalization. Performance in executive function was standardized based on normative data of the EpiTrack^®^ (689 healthy volunteers).

As demonstrated in Figure 4, the initial assessment (07.11.2011) showed deficits in executive functions and figural memory, which is indicative of frontal and right temporal dysfunction. The introduction of neuroleptic treatment, and adjunctive antiepileptic treatment with oxcarbazepine plus immunotherapy with methylprednisolone, was accompanied by significant deterioration in verbal and figural memory and executive function (second assessment: 07.02.2012). Within the next 5 months, verbal memory showed further significant decline, while figural memory and executive functions remained at the low-performance level (third assessment: 12.07.2012). About 9 months after the first 6-month cycle of Methylprednisolone (fourth assessment: 04.11.2013), verbal memory was found to be significantly improved and within the normal range, whereas figural memory and executive function remained unchanged. At the fifth assessment (25.07.2013) – after uptitration of Zonisamide as third concurrent antiepileptic agent – a general decline in all three cognitive domains (but especially in verbal memory) was observed. This fifth assessment also represented the baseline before introduction of basiliximab. The final neuropsychological evaluation (sixth assessment: 05.03.2014) after five cycles of basiliximab in combination with methylprednisolone revealed a steady decrease in executive function and again a significant decline in verbal memory. At this final follow-up, memory performance measured more than 3 SDs below the mean of the normative sample of healthy controls, while executive functions were more than 2 SDs below normal.

At disease onset, significant mood disorders and psychic disinhibition occurred simultaneously, with aggressive outbursts and physical violence, as described above. These changes diminished during therapy. As described in Table 1, the neuroleptic therapy with risperidone was discontinued under immunomodulatory therapy. However, risperidone was reduced from a daily dose of 2 mg down to 0.5 mg before therapy onset. The last contact with the patient took place 3 months after treatment ended, and he displayed significant affective disorders and psychic disinhibition again.

The visual EEG-analysis reported in Table 1 mostly revealed bitemporal alterations and did not show any obvious changes. Initially, there is a transition of main temporal focus from left to right that might coincide with volume changes in MRI (see MRI-Volumetry). In general, EEG-related biomarkers for LE are still missing.

### Flumazenil PET

**Figure 5:**
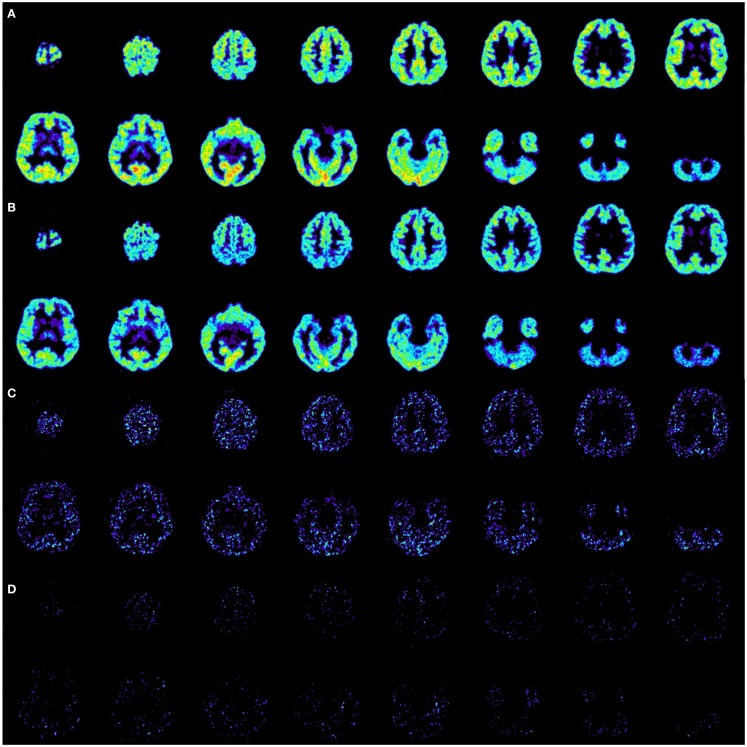
**Ethyl 8-fluoro-5-[(11)C]methyl-6-oxo-4H-imidazo[1,5-a][1,4]benzodiazepine-3-carboxylate positron emission tomography (**=**11C-flumazenil PET)**. **(A)** Scan before first application of basiliximab, normalized to injected activity of 580 MBq with an acquisition time from 20 to 40 min after injection. **(B)** Scan after sixth application of basiliximab, normalized to injected activity 674 MBq with the same acquisition time from 20 to 40 min after injection. **(C)** Subtraction A–B, only positive results are displayed. A decrease of activity from A to B is clearly visible. **(D)** Subtraction B–A, only positive results are displayed. An increase of activity from A to B is hardly distinguishable from random noise.

Before the first application and after the sixth application of basiliximab, an 11C-flumazenil PET (14) was performed at the nuclear research center in Jülich. Injected activity for the first scan was 580 and 674 MBq for the second scan. Data were normalized to the injected activity in order to compare both scans. Furthermore, the acquisition time was kept constant by starting the acquisition 20 min after the injection and stopping 40 min after the injection. Results are shown in Figure 5. Flumazenil uptake is reduced under therapy with basiliximab. Since flumazenil binds to GABA_A_ receptors, these findings can be interpreted as a reduction of GABA_A_-receptors under therapy, which might be a consequence of a better GABA – supply. Since GABAergic neurons contain glutamate decarboxylase enzymes (GAD, e.g., GAD65) to produce their transmitter, the observed reduction of GABA-receptors could be interpreted as a recovery of GABAergic neurons, thus implying preceding functional impairment by cytotoxic T-cells.

### Changes in seizure frequency

The seizure frequency before and during therapy was counted from the patient’s diary and is shown in Table 1. After 6 months, the seizure situation improved slightly: the duration became shorter and seizures occurred only at night. Since the anticonvulsant medication was kept constant for this period, these improvements could be attributed to the immune modulatory therapy. However, the patient’s significant weight-loss resulted in the decision to taper down Zonisamide. At the same time, the duration of the cortisone pulse therapy was reduced from 5 to 3 days. After these interventions, the number of seizures increased, as did their severity. The new anticonvulsant drug perampanel was given as an add-on. One month after reaching a constant daily dose of 10 mg, the seizures stopped for about 7 weeks. It remains unclear if this is the result of the long-lasting therapy with basiliximab or a success of the recently approved anticonvulsant drug perampanel, or perhaps a result of both. However, after stopping the immune modulatory therapy, even more seizures occurred (up to 5 GTC seizures per month, not shown in Table 1) and ultimately resulted in further changes regarding the choice of anticonvulsant drugs.

### MRI-volumetry

**Figure 6:**
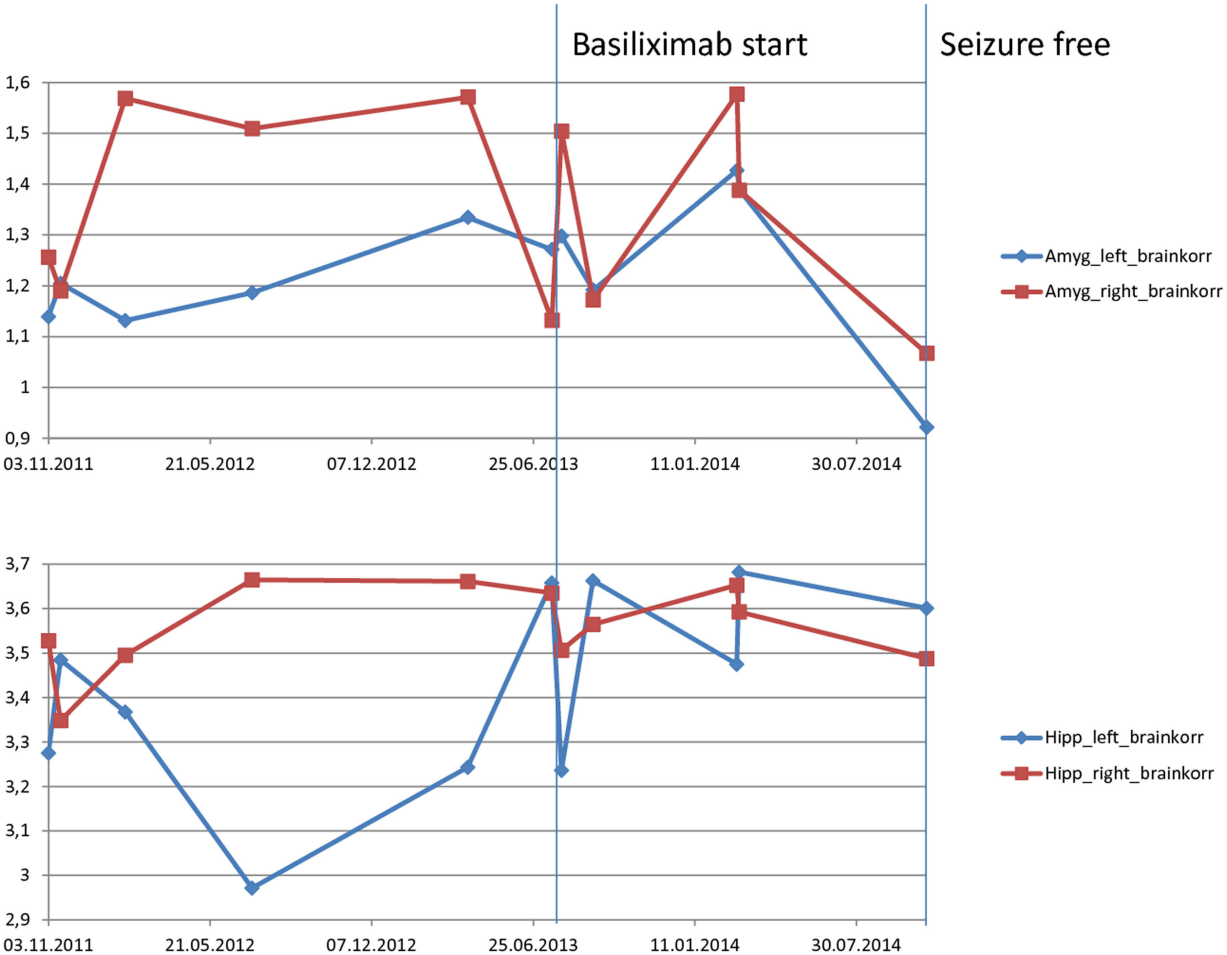
**Magnetic resonance imaging-volumetry of hippocampus and amygdala (measured in cubic millimeter) corrected by the whole brain volume (measured in cubic centimeter) of each scan**. The resulting values are unitless values measured as cubic millimeter per cubic centimeter. Before therapy, there is a fluctuation with more volume of right temporomesial structures. Last volumetry, acquired during the period without seizures shows a consensual volume-decrease in both amygdalae.

Between November 2011 and October 2014, a total of 11, 3-T MRI examinations were performed on the patient. Since it was difficult to schedule the investigations using only one MRI scanner, the investigations were performed either on a Siemens Trio (six times) or with a Philips Achieva Scanner, using a similar protocol. Volumetric analysis was performed on the comparable 3D, 1 mm^3^ isotropic voxels, MPR-T1-weighted image from each scanner. All image processing was implemented with the software FSL (Version 5.0.4, FMRIB Analysis Group, University of Oxford, England) (15). The analysis comprises whole brain segmentation (16) used to calculate the total brain volume, and subcortical segmentations of hippocampi and amygdala (17). As is usual in volumetric studies, raw measurements were corrected by the total brain volume, which aims to eliminate the variability between the two scanners. As seen in Figure 6, some fluctuations in the relative volume of right and left hippocampi and amygdalae were present throughout the observation period, which might coincide with a transition of main interictal temporal EEG-focus from left to right (Table 1). On the other hand, the volume of both amygdalae shows a noticeable reduction at the end of the observation period, when the patient was seizure free (Table 1).

### General side effects

About 2 weeks after the first application of basiliximab and methylprednisolone, the patient experienced renal failure for unknown reasons. He recovered completely while under infusion therapy and without the aid of dialysis. Since basiliximab is usually given during kidney transplantation and this side effect had not been previously described for basiliximab, it was assumed that the renal failure was not related to basiliximab. No other side effects occurred, besides a certain tiredness after the basiliximab infusion.

## Discussion and Concluding Remarks

Glutamate decarboxylase 65-ABs can also be found in other diseases, such as stiff person syndrome or even type 1 diabetes mellitus. However, the ABs from these entities do not differ in their target regions (18). In more than 90% of all cases with GAD65-ABs, there are GAD65-ABs against linear epitopes, which are only “visible” in fractions of GAD65, perhaps after lysosomal deconstruction, but not in the intact enzyme (19). In type 1 diabetes mellitus, an attempt was made to achieve immune tolerance against GAD65 by vaccinating with a GAD65 preparation. However, a phase 3 study was aborted because no improvements were found (20). In type I diabetes mellitus, a T-lymphocytic destruction of β cells is assumed to be the pathophysiological correlate (21). These experiences with GAD65-AB LE and other GAD65-AB-related diseases suggest that, in contrast to autoimmune encephalitis with ABs to neuronal surface antigens, additional factors may determine the type and course of GAD65-AB LE.

From other patients with a GAD-65-AB LE and with unilateral TLE who underwent amygdalohippocampectomy as epilepsy-surgical treatment, we know that cytotoxic T-lymphocytes were found in histological preparations of temporomesial structures (7). Experimental studies have shown that an attack of cytotoxic T-lymphocytes against neurons could result in a perforin-dependent electrical silencing, which is not necessarily linked to neuronal cell death (22). Thus, a chronic epilepsy by non-lethal cytotoxic attacks against GAD65 expressing GABAergic interneurons is possible. An alternative explanation for the existence of similar GAD65-ABs in different diseases could be that primarily there is an attack by cytotoxic T-lymphocytes against, e.g., GAD65 expressing GABAergic interneurons (or β-cells in type 1 diabetes mellitus) and second, partly denaturized GAD65 comes into contact with the humoral part of the immune system, resulting in the production of GAD65-ABs, also against linear epitopes of the enzyme, due to prior lysosomal denaturation. Therefore, a direct therapy against GAD65-ABs should fail, whereas an attenuated activation cytotoxic T-lymphocytes should be helpful.

The close correlation of the fraction of activated cytotoxic T-lymphocytes in CSF with clinical and paraclinical measures of disease activity in our case, together with the unambiguous response to basiliximab strongly argues in favor of a putative pathogenic role of cytotoxic T-cells in anti-GAD65 LE.

Overall, the treatment was not completely successful. The seizures became less severe during therapy, but seizure freedom was achieved only for a short time.

Neuropsychological tests did not show any definitive therapy-related changes; only affective disturbances and impulsiveness improved at a clinical level. MRI-volumetry showed, at most, a trend toward normalization of swollen amygdalae. Amygdala swelling is frequently observed in patients with LE.

Impressive results can be seen in the CSF. Here, the fraction of activated T-lymphocytes was normalized under treatment and, later on, also the AB concentration was reduced. Furthermore, impressive changes in GABA metabolism as represented by the flumazenil PET were observed. Unfortunately, only two PET scans exist because of problems related to radiation exposure. For the same reason, no control group of healthy persons was available as radioactive scans from healthy volunteers are difficult to obtain due to ethical reasons.

The clinical relapse at the end of the observation period might be caused by the formation of HADAs, a well-known complication of therapy with chimeric ABs (10, 11). Proving this hypothesis has not been possible up until now. The therapy, however, was discontinued in order to avoid anaphylactic reactions and, as a result, the clinical situation was even more impaired. The patient did not agree to an alternative therapy with natalizumab because of the risk of progressive multifocal leukoencephalopathy. We are anticipating the approval of a new, homologous therapeutic AB, which has shown to be effective in the long-term therapy of multiple scleroses (8, 23). This new AB, daclizumab hyp, is hypoallergenic and was administered in monotherapy in much higher doses (150 or 300 mg/month). The administered dose of 20 mg of basiliximab in this case was much lower. An increase might have been more effective, since the observed dramatic expansion of CD56 bright NK cells under Daclizumab hyp (23) could be observed here, but only a slight trend was visible (data not shown).

However, the dose of basiliximab was not increased as it has yet to be approved for administration at higher doses. On the other hand, the intervals of application could not be shortened since cortisone pulses are usually administered monthly. Because of security concerns, basiliximab was always given to the patient when he was on the ward during his in-hospital stay.

Based on this case report, a controlled study using a higher dose of daclizumab hyp for the therapy of GAD65-AB LE with increased fraction of cytotoxic T-lymphocytes in CSF might be reasonable.

## Conflict of Interest Statement

The authors declare that the research was conducted in the absence of any commercial or financial relationships that could be construed as a potential conflict of interest.
